# Toxic in vitro effects of flavoured-air inhalers containing essential oils in differentiated human nasal epithelial cells at air–liquid interface

**DOI:** 10.1007/s00204-026-04521-w

**Published:** 2026-08-12

**Authors:** Alexander N. Larcombe, E. E. Catchpole, P. Hopper, S. Stanley, A. Kicic, K. R. Landwehr

**Affiliations:** 1 Occupation, Environment and Safety, School of Population Health, Curtin University, GPO Box U1987, Perth, WA 6845 Australia; 2 Wal-yan Respiratory Research Centre, The Kids Research Institute Australia, Nedlands, Australia; 3 WA Organic and Isotope Geochemistry Centre, School of Earth and Planetary Sciences, Faculty of Science and Engineering, Curtin University, Bentley, WA Australia; 4 Department of Respiratory and Sleep Medicine, Perth Children’s Hospital, Nedlands, WA Australia; 5 Centre for Cell Therapy and Regenerative Medicine, School of Medicine and Pharmacology, The University of Western Australia and Harry Perkins Institute of Medical Research, Perth, WA Australia

**Keywords:** Flavoured-air, Essential oils, Airway epithelial cells, Air–liquid interface, Respiratory toxicity

## Abstract

Flavoured-air inhalers containing essential oils are marketed as safe, natural alternatives to smoking and vaping, yet their potential respiratory toxicity remains effectively unexamined. Manufacturers claim safety based on "100% natural" ingredients, despite evidence that essential oil inhalation can cause adverse respiratory effects. Our objective was to assess the in vitro toxicity of six commercially available flavoured-air products on human nasal airway epithelial cells. Differentiated air–liquid interface cultures of primary nasal airway epithelial cells from eight healthy adults were exposed for one hour to air (control) or flavoured air from three devices (Füm, Lio, Cigtrus; two flavours each) generated by drawing air through essential oil-infused cores at a continuous airflow of 1 L/min for one hour into an 1000 mL exposure chamber. Outcomes included transepithelial electrical resistance, cytotoxicity, protein release, cytokine/chemokine profiling and histological assessment. Chemical analysis characterised the chemical composition of device cores and identified 29 compounds across the six products, including the possible carcinogen pulegone and cytotoxic cinnamaldehyde. Five of six flavours significantly reduced transepithelial electrical resistance indicating compromised epithelial barrier integrity. Mediator responses were compartment-specific: apical surfaces showed predominantly pro-inflammatory profiles while basal release was broadly suppressed. Histological analysis revealed reduced cilia coverage, fewer mucus-producing cells, and decreased epithelial thickness across multiple treatments. This study shows that a single one-hour exposure to flavoured-air vapour led to epithelial barrier dysfunction, immune dysregulation, and structural damage in healthy human nasal airway epithelial cells, contradicting manufacturer safety claims. Rigorous toxicological evaluation of these products is urgently needed.

**Supplementary Information:**

The online version contains supplementary material available at https://doi.org/10.1007/s00204-026-04521-w.

## Introduction

Flavoured-air devices, such as Füm, Lio and Cigtrus, are a relatively new category of product designed to facilitate the inhalation of essential oils (Tackett and Rebuli 2022). They are often advertised as “effective at helping stop bad habits” and as smoking/vaping cessation tools and are thus targeted at smokers and vapers. Their efficacy in this context has not been adequately assessed, although manufacturers use this as a marketing strategy. For example, an unpublished study, conducted by an unspecified third-party on behalf of Füm, found that in a random sample of 400 Americans, flavoured air was approximately twice as effective for smoking and vaping cessation compared with nicotine replacement therapy (Füm 2024). Similarly, Lio use is claimed to make 100% of people “more in control of urges” (Lio 2024), while Cigtrus is advertised as a “natural smoking alternative” (Cigtrus 2024). While there is limited, subjective, evidence that inhalation of some essential oils may reduce nicotine cravings in the short-term (Arslan Işık and Bilgin 2022; Cordell and Buckle 2013; Rose and Behm 1994), the cessation claims of flavoured-air inhaler manufacturers primarily focus on addressing hand-to-mouth habits and oral fixation (Jarry 2024). This is presumably because many flavoured-air devices do not contain essential oils previously associated with reductions in cravings.

Importantly, manufacturers also market them using phrases such as “all natural”, “no chemicals” and “no-nicotine”. The pitch is that because the essential oils used in them are “100% natural”, these products are safe for inhalation use. Indeed, many essential oils are known for their antibiotic, antioxidant and anti-inflammatory properties (reviewed in Valdivieso-Ugarte et al. 2019), suggesting that flavoured-air inhalers may have beneficial health impacts in certain situations. This, however, is not the complete picture, as there is also considerable evidence showing that inhalation of essential oils can also have significant toxic effects including endocrine disruption leading to developmental abnormalities, neurotoxicity and adverse cardiopulmonary effects (Diaz et al. 2016; Lee et al. 2022; Tisserand and Young 2013). From a respiratory health point of view, it is known that essential oil inhalation can be harmful (Kodama et al. 2022), and it is not only when the oils are used with a diffuser. A recent case-report detailed how daily use of a Füm inhaler led to the development of acute eosinophilic pneumonia in an otherwise healthy 49-year-old woman (Hernandez and Foley 2024).

Taken together, these findings establish a clear toxicological concern: flavoured-air devices deliver volatile compounds directly to the airway epithelium via inhalation. Unlike diffuser-based aromatherapy, where exposure is ambient and intermittent, these devices are designed for direct, concentrated oral inhalation, creating a potentially more hazardous exposure scenario. The airway epithelium is the first tissue to encounter these compounds, and its integrity is critical for mucociliary clearance, immune defence, and barrier function against pathogens and allergens. Despite this, no independent study has characterised the effects of flavoured-air device exposure on airway epithelial cells using a physiologically relevant model. Therefore, we conducted a study to assess health effects resulting from flavoured air inhaler exposure in vitro. Nasal airway epithelial cells (AEC) from healthy adults, grown into realistic air–liquid interface in vitro airway models were exposed to flavoured air from a variety of devices prior to assessment of a range of relevant health outcomes. We hypothesised that exposure would result in negative respiratory health effects that would vary between flavours.

## Methods

### Ethics and participants

Primary nasal airway epithelial cells were collected by nasal brushing the inferior turbinate of 8 non-asthmatic adults aged 20–30 years (4 male) from the CONQUER cohort study (RGS0000004379). Low-risk Eethical approval was obtained from the Curtin University Human Research Ethics Committee (HRE2025-0175).

### Chemical analyses of flavoured-air

Essential oil-infused cores from six commercially available flavoured-air devices (two flavours each of Füm, Lio and Cigtrus) were analysed by gas chromatography–mass spectrometry (GC–MS). Fluid was recovered from each core, diluted to 1 mg/mL in HPLC-grade methanol, and analysed on an Agilent 7890A/5975C system with an HP-INNOWax column. Compounds were identified by spectral matching against the NIST 2017 library, and relative proportions calculated from integrated peak areas. Full instrument parameters are provided in Online Resource 1.

### Tissue culture

Primary nasal AECs were expanded and seeded onto Transwell inserts (12 mm, 0.4 μm pore) in PneumaCult ExPlus, then air-lifted and differentiated in PneumaCult ALI medium for at least 28 days (Martinovich et al. 2017). Additional details are described in Online Resource 1. Transepithelial electrical resistance (TEER) was monitored weekly. Each donor’s cells were exposed to all seven conditions (medical air control and the six flavoured-air products) in a fully crossed design, with duplicate inserts generated per donor per condition (up to 16 inserts per treatment). Cultures with pre-exposure TEER deviating substantially from the donor mean were excluded; in addition, one donor’s post-exposure TEER measurements were excluded as poor quality, so TEER was based on seven donors. Final culture numbers were n = 16 per treatment for BCA protein, n = 15–16 for LDH, n = 14–16 for mediators, n = 13–14 for TEER, and n = 6 per treatment for histology (two donors excluded for poor-quality sections); the full breakdown is given in Online Resource 2.

### Exposure protocol

**Fig. 1:**
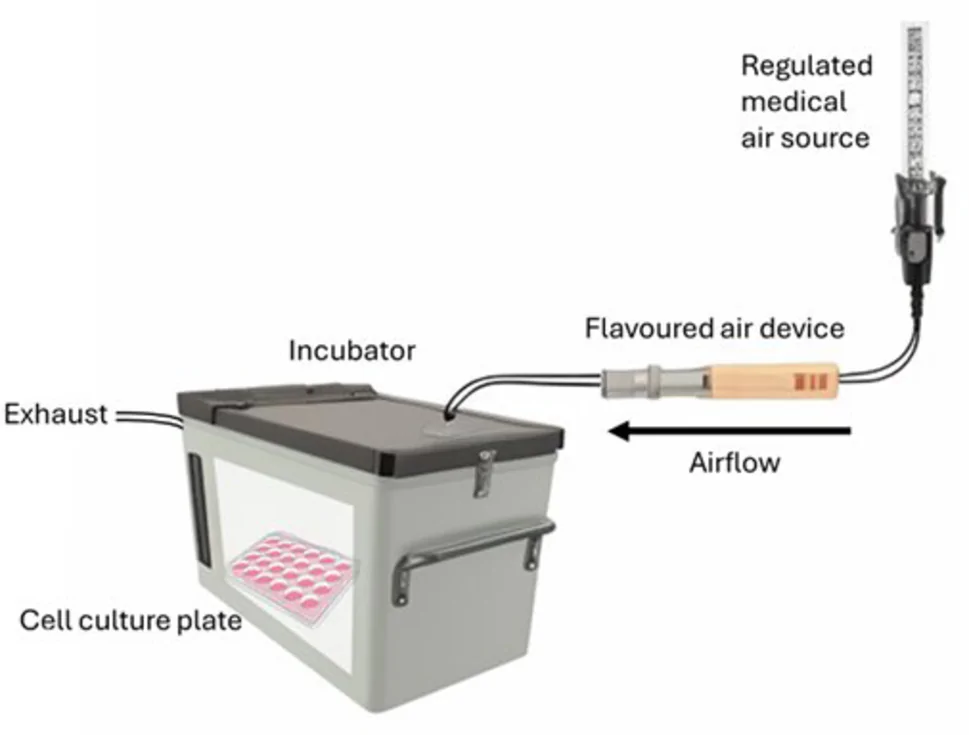
Schematic of the experimental setup. Medical air flowed through the flavoured-air devices at 1 L/min, into an 1000 mL exposure chamber containing the ALI-culture plates

Three flavoured-air devices were tested with two flavours each: Füm Crisp Mint (FCM) and Maple Pepper (FMP); Lio Minty Magic (LMM) and Cinnamon Spice (LCS); and Cigtrus Icy Peppermint (CIP) and Grapefruit (CGF). All devices were purchased new from online suppliers. ALI cultures were exposed for one hour to either medical air (control) or flavoured air (FA) generated by passing medical air at 1 L/min through each device and into 1000 mL exposure chambers maintained at 37 °C (Fig. 1). The one-hour duration was selected to approximate a plausible period of cumulative daily use, as manufacturers recommend repeated sessions throughout the day to manage cravings. No published usage topography data exist for flavoured-air devices. Future studies incorporating varied exposure durations and intermittent delivery would enable dose–response evaluation. After exposure, cultures were returned to standard incubation conditions (37 °C, 5% CO_2_/95% air).

### Lactate dehydrogenase and protein

Twenty-four hours post-exposure, TEER was measured and apical wash and basal supernatant were collected. Cytotoxicity was assessed by lactate dehydrogenase (LDH) release (CytoTox 96, Promega) and protein concentration by BCA assay (Pierce, Thermo Fisher Scientific) (Looi et al. 2016).

### Mediators

Mediator levels were measured in apical and basal compartments 24 h post-exposure using a 27-plex human cytokine panel (Bio-Rad). Data were processed using custom Python software to remove outliers (> 3 SD) and replace values below or above the limits of detection (see Online Resource 1 for details).

### Histology

Inserts were fixed in 10% formalin, embedded in paraffin, sectioned (5 μm) and stained with Alcian Blue/Neutral Red. Sections were scanned at 40× magnification (SLIDEVIEW VS200, Olympus) and analysed using QuPath v0.6.0. Outcomes included the number and area of mucus-producing cells normalised to basement membrane length, epithelial thickness at three standardised points, cell layer count and cilia density score (1–3 scale). Two patient samples were excluded due to poor-quality sections.

### Statistics

Data are presented as mean ± standard deviation. All outcomes were analysed using linear mixed-effects models with treatment as a fixed effect and patient as a random intercept. Where models did not converge, alternative model structures were tested (see Online Resource 1). Pairwise comparisons were derived from fitted model estimates. As all comparisons were derived from a single model with shared variance structure, no additional correction for multiple comparisons was applied. Significance was set at p < 0.05. Two sensitivity analyses were performed for the primary endpoints and are reported in Online Resource 3: (i) a nested model with flavour nested within brand (patient as a random intercept), and (ii) inclusion of sex as a fixed covariate together with a sex x treatment interaction.

To explore which chemical constituents may drive biological responses, redundancy analysis (RDA) and Spearman rank correlation analysis were used to relate the semi-quantitative GC–MS chemical profiles of the six products to 47 biological endpoints (TEER, LDH, BCA, Bio-Plex cytokine/chemokine concentrations above the limit of detection, and six histological parameters). Full methodological details are provided in Online Resource 1.

### LLM/AI use

An AI language model (Claude, Anthropic) was used to assist with data visualisation, figure preparation, and manuscript editing. All AI-generated content was reviewed, verified, and revised by the authors, who take full responsibility for the accuracy and integrity of the final manuscript.

## Results

### Chemical analyses

Twenty-nine different chemicals were detected in the flavoured-air device cores, and there was one compound we couldn’t identify. Each variety contained between six (CGF) and 20 chemicals (FMP). The most abundant chemical detected was menthol, which was detected in all six flavours (15.6 to 95.0% relative abundance). The only other analyte found in all six flavours was caryophyllene. The distribution of individual chemicals across the six products, and the resulting clustering of products by shared composition, is shown in Online Resource 4. The two Cigtrus products had the simplest profiles, and the two mint-dominated products (FCM and LMM) clustered together, separately from the spice-type products. A complete list of chemicals detected is included in Online Resource 5. Detections of note include pulegone (IARC Group 2B: possible carcinogen) which was found in 4 of 6 cores, and cinnamaldehyde (cytotoxic to lung cells and banned in e-liquids in Australia), which was found in 2 of 6 cores. Fourteen of the 29 chemicals detected are classified as skin sensitisers (GHS H317) and thirteen are classified as skin/eye irritants (GHS H315, H318 or H319).

### LDH and protein release

**Fig. 2:**
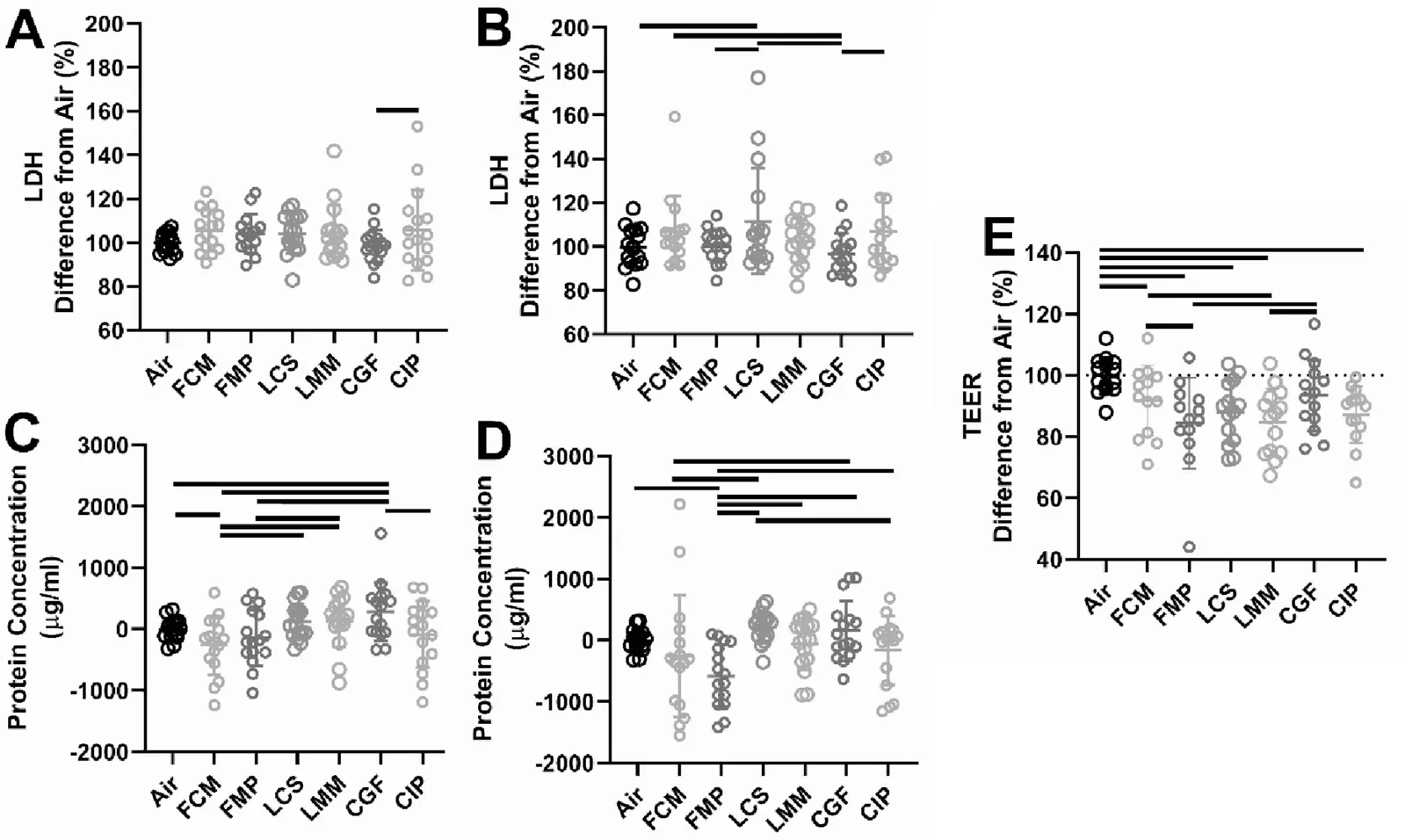
Lactate dehydrogenase in apical (**A**) and basal (**B**) compartments presented as percent difference from Air. Protein concentration in the apical (**C**) and basal (**D**) supernatant, and transepithelial electrical resistance (TEER; **E**) in ALI cultures exposed to Air or flavoured air (FCM = Füm Crisp Mint, FMP = Füm Maple Pepper, LMM = Lio Minty Magic, LCS = Lio Cinnamon Spice, CIP = Cigtrus Icy Peppermint and CGF = Cigtrus Grapefruit). Black bars represent significant differences between treatments (p < 0.05). Dots represent individual data points (2 per patient), with mean ± SD. Note different scales

Lactate dehydrogenase (LDH) and protein (BCA) levels were assessed as patient-corrected percentage difference from Air control (i.e. Air control was normalised to 100%) and measured in the apical and basal supernatant. For apical LDH (Fig. 2A), CIP was significantly higher than CGF (p = 0.049), but there were no other significant differences (p > 0.050 in all cases). In the basal compartment (Fig. 2B), LCS exposed cells had significantly higher LDH than Air (p = 0.020), but there were no other significant differences between treatments and Air (p > 0.117 in all cases). LDH was between 96.6 and 111.5% of Air for all treatments.

There were significant effects of flavoured air exposure on protein levels in the apical compartment (Fig. 2C). CGF exposed cells had significantly more protein compared with Air (p = 0.031), FCM (p < 0.001), FMP (p = 0.001) and CIP (p = 0.005). FCM exposed cells had significantly less protein compared with LCS (p = 0.004) and LMM (p = 0.003). FMP exposed cells also had significantly less protein compared with LMM (p = 0.043). In the basal compartment (Fig. 2D), FMP exposed cells released significantly less protein compared with Air (p < 0.001), CGF (p < 0.001), LCS (p < 0.001) and LMM (p = 0.003). CGF exposed cells had significantly more protein compared with FCM (p = 0.019) and CIP (p = 0.031). LCS also had significantly more protein compared with CIP (p = 0.031) and FCM (p = 0.007).

### Permeability

Permeability was assessed as patient-corrected percentage difference from Air control (i.e. Air control was normalised to 100%; Fig. 2E). Flavoured air exposure resulted in significant decreases in TEER for FCM, CIP, FMP, LCS and LMM compared with Air (p < 0.027 in all cases). For these exposures, TEER was 84.4 to 91.8% of Air. CGF was the only flavoured air treatment that did not significantly differ from Air (p = 0.065). CGF exposed cells had significantly higher TEER than FMP (p = 0.010) and LMM (p = 0.015). FCM exposed cells had significantly higher TEER than FMP (p = 0.034) and LMM (p = 0.049). These product-level effects were robust to a nested (flavour-within-brand) model, in which all three brands significantly reduced TEER (Füm: -11.9%, Lio: -13.6%, Cigtrus: -9.7%; all p ≤ 0.002) and within-brand flavour differences remained significant for four of the five primary endpoints, confirming that the two flavours of a brand should not be pooled (Online Resource 3).

### Mediators

Mediator levels were measured in both apical and basal supernatant (Online Resources 6 and 7). In apical supernatant, 16 (out of 27) analytes were released above the limit of detection in at least 70% of measurements. Analytes where > 30% of measurements were out of range were excluded from further analyses (IL-1β, IL-2, IL-4, IL-10, IL-12(p70), IL-13, IL-15, IL-17, FGF basic, PDGF-bb and VEGF). In the basal supernatant, 20 (out of 27) analytes were released above the limit of detection in at least 70% of measurements. Again, analytes where > 30% of measurements were out of range were excluded from further analyses (IL-1β, IL-2, IL-7, IL-10, IL-12(p70), IL-13, IL-15).

**Fig. 3:**
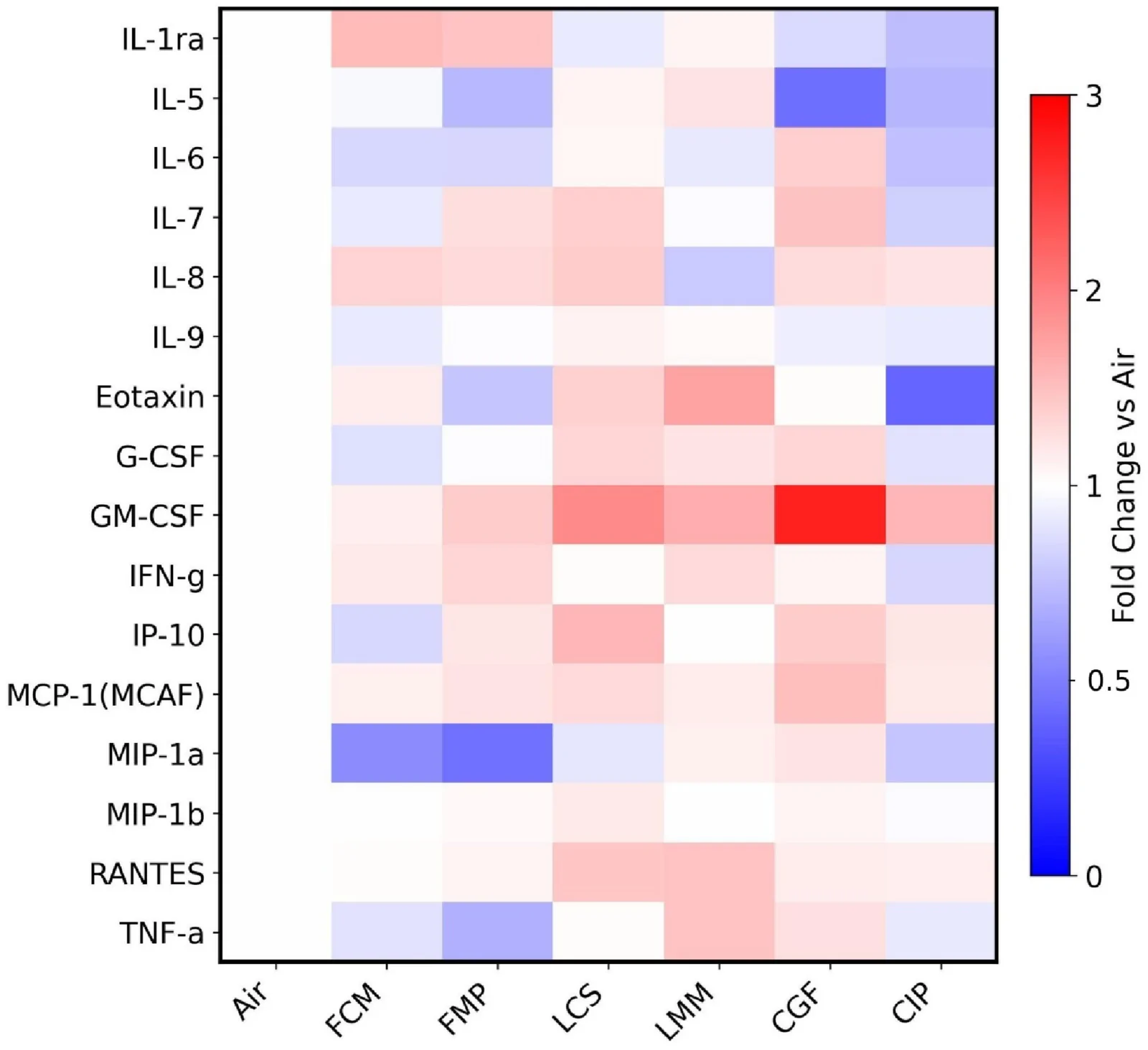
Fold change (vs Air) of mediators in the apical supernatant. Data are per-patient corrected. FCM = Füm Crisp Mint, FMP = Füm Maple Pepper, LMM = Lio Minty Magic, LCS = Lio Cinnamon Spice, CIP = Cigtrus Icy Peppermint and CGF = Cigtrus Grapefruit

**Fig. 4:**
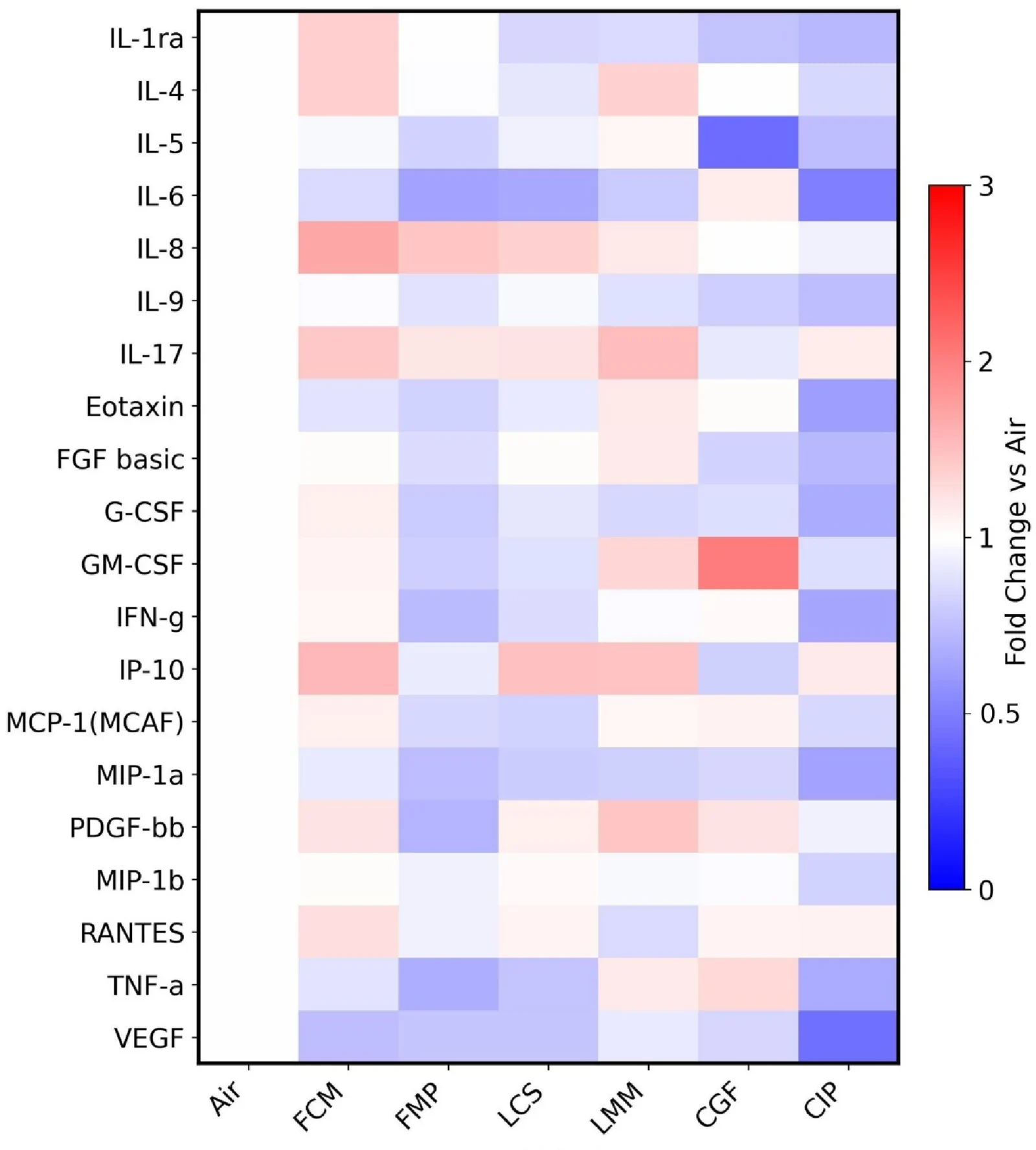
Fold change (vs Air) of mediators in the basal supernatant. Data are per-patient corrected. FCM = Füm Crisp Mint, FMP = Füm Maple Pepper, LMM = Lio Minty Magic, LCS = Lio Cinnamon Spice, CIP = Cigtrus Icy Peppermint and CGF = Cigtrus Grapefruit

There were complex effects of exposure for both apical (Fig. 3, Online Resource 6) and basal supernatant (Fig. 4, Online Resource 7). There was an overall pattern of increased levels of most mediators in the apical compartment, and decreased levels of most mediators in the basal compartment after flavoured air exposure.

In the apical compartment FCM and CIP exposures demonstrated largely immunosuppressive characteristics, with significantly reduced release of many mediators compared with Air control, including MIP-1α (FCM) and IL-5 (both). Conversely, LCS, LMM and CGF elicited more robust pro-inflammatory profiles, including increased release of IP-10, GM-CSF, IL-7, RANTES and TNF-α. Overall, CGF exposure led to the most robust inflammatory response. There was no statistically significant effect of treatment on a small proportion of mediators in the apical compartment (G-CSF, IFNγ and MIP-1β).

Compared with the apical compartment, basal mediator release was markedly attenuated, with most exposures resulting in reduced mediator release compared with Air control. There was the consistent suppression of several mediators across flavoured air treatments, particularly IL-5, MIP-1α and VEGF, which exhibited reduced release across all exposures. Air control demonstrated the highest levels for multiple mediators including IL-5, VEGF, and FGF basic. FMP exposure produced the most consistent downregulation pattern, with significant reductions in most mediators including IL-4, IL-5, TNF-α, VEGF, and PDGF-bb. FCM exposure resulted in modestly increased release of most mediators while CIP exposure produced the most severe suppressive profile, with significant reductions across most mediators. There was no statistically significant effect of treatment on a small proportion of mediators in the basal compartment (IL-6, MCP-1, MIP-1β and RANTES).

### Histology

**Fig. 5:**
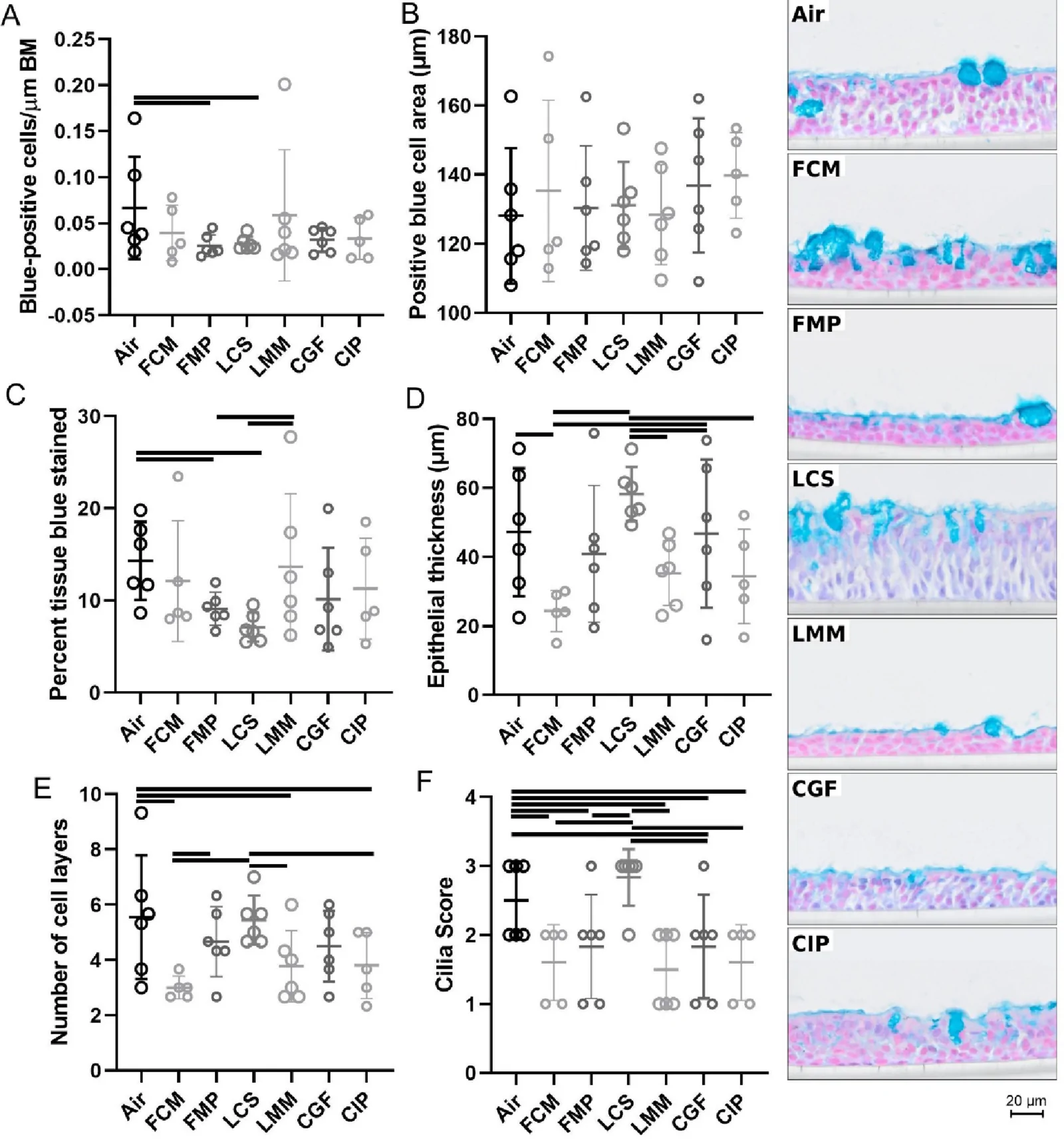
Histology measures, and representative images, of ALI cultures exposed to Air, or flavoured air (FCM = Füm Crisp Mint, FMP = Füm Maple Pepper, LMM = Lio Minty Magic, LCS = Lio Cinnamon Spice, CIP = Cigtrus Icy Peppermint and CGF = Cigtrus Grapefruit). Black bars represent significant differences between treatments (p < 0.05). Dots represent individual data points (1 per patient), with mean ± SD. Note different scales

Flavoured air exposure had a range of significant impacts on ALI structure (Fig. 5). FMP and LCS exposed cells had fewer blue-positive stained cells per unit length of basement membrane (p < 0.049 in both cases; Fig. 5A), but there was no significant difference in the average size of blue-positive stained cells between treatments (p > 0.18 in all cases; Fig. 5B). The fewer blue-positive stained cells in FMP and LCS transferred to a significantly lower amount of blue-stained tissue in those treatments (as a percentage of total tissue area) compared with Air (p < 0.021 in both cases; Fig. 5C). The same two treatments also had a significantly lower amount of blue-stained area compared with LMM (p < 0.042 in both cases; Fig. 5C). The thickness of the epithelium also varied between treatments (Fig. 5D), with LCS exposed cultures being significantly thicker than FCM, FMP, CIP and LMM (p < 0.022 in all cases). LCS was not significantly different to Air (p = 0.145). FCM exposed cultures were significantly thinner than Air, CGF and LCS (p < 0.008 in all cases). Unsurprisingly, epithelial thickness was reflected in the mean number of cell layers, which varied similarly across treatments (Fig. 5E). Air controls and LCS had significantly more cell layers than CIP, FCM and LMM (p < 0.035 in all cases). FCM was again a standout, with fewer cell layers compared with Air, FMP and LCS. Finally, cilia score was also impacted by exposure (Fig. 5F), with all flavoured air treatments except LCS having a lower score than Air (p < 0.042 in all cases). LCS exposed cells also had a higher score than all other flavoured air treatments (p < 0.002 in all cases).

### Chemical–biological associations

**Fig. 6:**
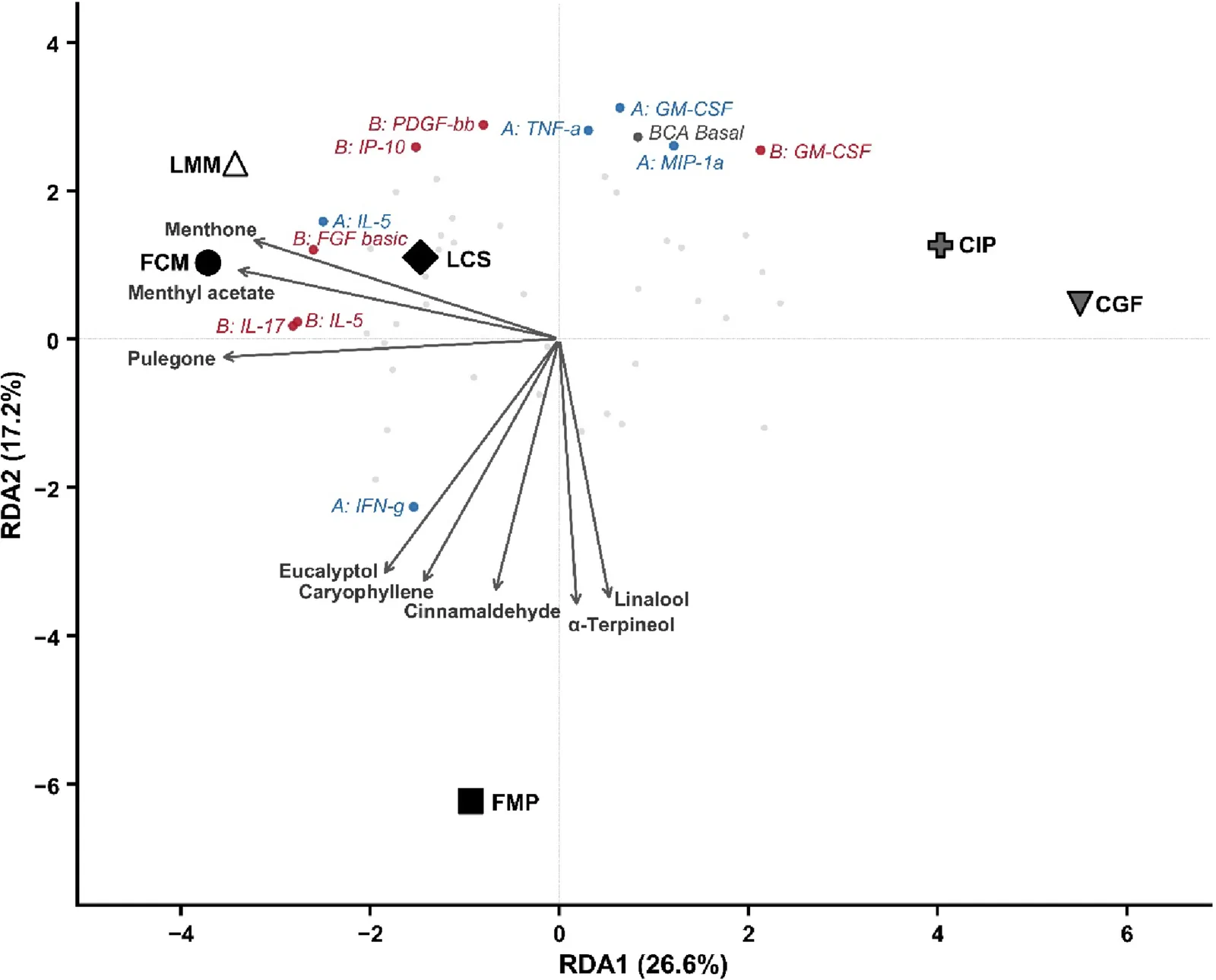
Redundancy analysis (RDA) triplot relating chemical composition to biological responses across six flavoured air products. The RDA was constrained by the first two principal components of the chemical composition matrix (12 analytes present in ≥ 2 products), which captured 87.2% of chemical variance. The response matrix comprised 47 biological endpoints. Product positions reflect their overall biological response profiles as predicted by chemical composition. Arrows indicate the direction and strength of association of chemical analytes with the RDA axes. Small dots represent individual biological endpoints, with the 12 most strongly loaded endpoints labelled: blue, apical mediators; red, basal mediators; dark grey, barrier/toxicity measures. Unlabeled grey dots represent the remaining 35 endpoints. The model explained 43.8% of total biological variance (RDA1: 26.6%, RDA2: 17.2%; permutation test F = 1.17, p = 0.235, 9,999 permutations). FCM = Füm Crisp Mint; FMP = Füm Maple Pepper; LCS = Lio Cinnamon Spice; LMM = Lio Minty Magic; CGF = Cigtrus Grapefruit; CIP = Cigtrus Icy Peppermint. A = apical; B = basal

RDA explained 43.8% of biological variation (R^2^ = 0.438, adjusted R^2^ = 0.064) but did not reach global significance (permutation p = 0.235), the large discrepancy between R^2^ and adjusted R^2^ reflecting overfitting due to the high predictor-to-observation ratio (Fig. 6). Pairwise Spearman correlations identified 41 significant (p < 0.05) chemical–biological associations. Pulegone showed the strongest association, correlating with basal IL-17 (r_s_ = 0.986, p < 0.001). A cluster of co-varying mint terpenoids (menthone, menthyl acetate, isomenthone) was associated with elevated basal IL-5 and IL-17 and suppressed MCP-1 in both compartments. Caryophyllene oxide showed an opposing pattern, correlating negatively with apical IL-6, G-CSF, GM-CSF, TNF-α, and MIP-1α (all rₛ =  − 0.845, p = 0.034; identical coefficients reflecting rank ties at n = 6). Cinnamaldehyde was associated with reduced goblet cell density (r_s_ =  − 0.845, p = 0.034). TEER and apical LDH showed no significant chemical associations.

## Discussion

This study demonstrates that short-term exposure to the vapour output from flavoured-air devices can result in significant toxicity in differentiated human nasal airway epithelial cells, with effects varying between products and flavours. Exposure resulted in reductions in TEER, complex compartment-specific changes in mediator production, and a range of histological impacts. These outcomes challenge industry marketing claims that these products are safe because they contain "100% natural" ingredients.

A key finding was the consistent reduction in TEER compared with Air, observed across five of six flavoured-air treatments (FCM, CIP, FMP, LCS, LMM; ~ 8–16% reduction). There was also a non-statistically significant reduction in TEER for CGF (~ 7% reduction compared with Air). Reductions in TEER are commonly measured in ALI culture models post exposure to aerosolised insults such as cigarette smoke (Olivera et al. 2007), e-cigarette aerosol (Raduka et al. 2023) and particulate matter (Landwehr et al. 2022). They can be interpreted as compromised barrier integrity, with epithelial barrier dysfunction being a hallmark of respiratory diseases including asthma and COPD (Georas and Rezaee 2014; Hackett 2012). Compromised barrier integrity can facilitate translocation of inhaled pathogens, allergens, and pollutants across the epithelium potentially increasing their access to subepithelial immune and structural cells and increasing susceptibility to viral or bacterial infection (Yuksel and Turkeli 2017). This outcome is important as it shows that despite the antimicrobial properties of some essential oils, exposing the airway epithelium directly to their vapour negatively impacts epithelial integrity. Experiments which expose ALI cultures to other aerosolised insults (e.g. a respiratory virus) post exposure to flavoured-air are required to more fully elucidate this outcome.

Mediator production post exposure was complex and varied considerably between the apical and basal compartments. In the apical compartment, most flavoured-air exposures led to increased levels of pro-inflammatory mediators including GM-CSF, IL-8, MCP-1 and RANTES, with CGF producing the most robust inflammatory response. These increases may be representative of direct toxicity at the luminal surface, and initiation of luminal defence mechanisms, as seen after e-cigarette aerosol exposure (Masso-Silva et al. 2021). These mediators are all pro-inflammatory and their increased production would result in recruitment/activation and enhanced survival of immune cells such as eosinophils, neutrophils and macrophages (Bezerra et al. 2023). Higher levels of these cells in the airways amplify local inflammation and tissue damage and could lead to potential remodelling after chronic exposure (Britt et al. 2023). IFNγ was also relatively consistently increased (not statistically significantly) in the apical supernatant post flavoured-air exposure. This is a critical host defence mechanism, but also drives chronic inflammation, tissue damage, and promotes Th1-mediated immune responses in the airways (Ramana et al. 2015).

Conversely, basal mediator release was markedly suppressed, with consistent downregulation of many mediators including IL-5, IL-6, MIP-1α, and VEGF across most treatments. FMP and CIP resulted in the greatest suppression. A polarised mediator response to aerosolised insults has been reported before and illustrates the distinct biological and functional domains of the airway epithelium (He et al. 2021; Skronska-Wasek et al. 2021). Reduced basal mediator levels could be interpreted as systemic immunosuppression and reduced signalling to underlying immune cells, potentially increasing susceptibility to infections and impairing anti-tumour mechanisms (Baldassi et al. 2021; Kountz et al. 2022; Liu et al. 2023). For example, IL-5 downregulation would result in blunted systemic eosinophil activation, survival, and recruitment, weakening type 2 immune responses, and potentially contributing to poor asthma control (Takatsu 2011). Reduced systemic levels of VEGF could delay tissue healing and impair tumour growth suppression (Lee et al. 2025). Combined, these outcomes indicate that flavoured-air exposure has the potential to significantly impact both local and systemic immune responses.

Flavoured-air exposure also resulted in significant structural alterations to the ALI cultures, particularly reduced mucus in two “spice” type flavours (FMP and LCS) exposed cells and altered epithelial thickness and cilia coverage across treatments. Although it is a relatively crude measure, the reduction in cilia score, compared with Air, observed for all treatments except LCS is concerning. Reduced presence/activity of cilia would impair mucociliary clearance, which is a critical innate defence mechanism against inhaled insults (Knowles and Boucher 2002). Combined with the immune dysregulation seen post-exposure, this change could further predispose users to respiratory infections and reduced airway clearance of pollutants, allergens, and other pathogens. We also measured reductions in the number of cell layers (compared with Air) in three flavoured-air treatments, which was partially recapitulated in absolute epithelial thickness. These changes suggest that flavoured-air exposure led to structural injury of sufficient severity to damage or kill epithelial cells. They are also consistent with the reductions in TEER seen post-exposure, again indicating compromised epithelial barrier integrity as seen in e-cigarette aerosol exposed cells (Gandhi et al. 2025).

Notably, LCS produced a distinct profile compared to other treatments whereby epithelial thickness and cilia coverage were not significantly different to Air control, although it still induced a robust apical inflammatory response and TEER was reduced. This suggests that toxicity profiles are flavour-dependent, likely reflecting differences in essential oil composition and concentration between products.

Chemical analyses revealed a complex mixture of volatile and semi-volatile organic compounds in the flavoured-air device cores. Unsurprisingly, many of the compounds detected were monoterpenes or sesquiterpenes, which are typically extracted from plants and are found extensively in essential oils (Loza-Tavera 1999). These compounds are known for their high volatility, pleasant aromas, and diverse therapeutic properties, including anti-inflammatory and antimicrobial effects (Zuo et al. 2020). It is feasible that these compounds had beneficial impacts on ALI-culture health, however the predominance of menthol across all flavours (15.6 to 95.0% relative abundance) warrants particular attention. Menthol is added to cigarettes (Willis et al. 2011) and e-liquids (Rosbrook and Green 2016) to mask the harshness of inhaled irritants, potentially facilitating deeper inhalation and greater exposure to toxic substances. There is also evidence that menthol exposure increases oxidative stress and levels of inflammatory cytokines in ALI-cultures (Nair et al. 2020), potentially leading to respiratory disease.

Notably, cinnamaldehyde was detected in two of the six flavours (relative abundance 6.93% in LCS and 20.0% in FMP). Recent studies demonstrate that cinnamaldehyde exposure impairs ciliary beat frequency in human bronchial epithelial cells by disrupting mitochondrial function and reducing intracellular ATP levels (Clapp et al. 2019). Furthermore, cinnamaldehyde has been shown to suppress respiratory innate immune cell function, including neutrophil and alveolar macrophage phagocytosis, which aligns with the complex immunomodulatory effects we observed (Clapp et al. 2017).

Also concerning was the detection of pulegone in four of the six flavoured-air products, albeit at very low levels (0.48 to 0.98% relative abundance). Pulegone has been classified as possibly carcinogenic to humans (Group 2B) by the International Agency for Research on Cancer (IARC 2016) and, based on carcinogenicity findings in rodent bioassays, synthetic pulegone was removed from the list of permitted food flavouring additives by the US Food and Drug Administration in 2018. While the Group 2B ranking of pulegone is based on ingestion exposure, and inhalation data are not available, studies suggest that inhalation of pulegone in e-cigarette aerosol raises significant health concerns (Jabba and Jordt 2019). The combination of pulegone with other detected chemicals classified as skin sensitisers (n = 14) and skin/eye irritants (n = 13) suggests that chronic exposure to these flavoured-air products may result in cumulative health effects beyond those captured in our acute exposure model.

The RDA triplot revealed that product clustering was largely brand-dependent, with both Lio and both Cigtrus products grouping closely together, suggesting that within-brand formulations share a common chemical base that drives similar biological responses regardless of flavour variant. In contrast, the two Füm products (Crisp Mint and Maple Pepper) were widely separated in ordination space, consistent with their distinct terpene profiles and indicating that flavour category (mint *vs* spice) can be a stronger determinant of biological outcome than brand identity. The correlation between pulegone and basal IL-17 is notable given that pulegone bioactivates to the hepatotoxic metabolite menthofuran. IL-17 drives airway neutrophilia (Ferretti et al. 2003) and mucus hypersecretion via goblet cell metaplasia (Pezzulo et al. 2019). The mint terpenoid–inflammatory signature (elevated IL‑5 and IL‑17 with suppressed MCP‑1) seen in our study is consistent with type 2/Th17 immune activation. Caryophyllene oxide’s anti‑inflammatory profile aligns with the documented CB2 receptor agonism effects of β‑caryophyllene (a closely related sesquiterpene), which may have a role in regulation of airway barrier integrity and wound healing in epithelial cells (Wang et al. 2016; Wright et al. 2005) and further, indicates that not all essential oil constituents produce uniformly harmful effects. Cinnamaldehyde is a known TRPA1 agonist (Alpizar et al. 2013); TRPA1 overactivation can cause epithelial stress and cytotoxicity (Nguyen et al. 2020), which may underlie the reduced goblet cell density in this dataset.

There are several strengths and weaknesses to this study which should be acknowledged. A key strength is that (to the best of our knowledge), this is the first independent study to assess the health impacts of flavoured-air exposure in vitro. Our outcomes were made more relevant by exposing primary human nasal airway epithelial cells at the air–liquid interface directly to flavoured-air from popular devices and a variety of flavours. That said, our study employed an in vitro exposure protocol, which does not fully recapitulate an in vivo exposure (including repeated dosing and systemic effects). We chose a one-hour exposure as no published usage topography data exist for flavoured-air devices. Future studies could incorporate a range of exposure periods to enable dose-responses to be evaluated. We also used nasally-derived airway epithelial cells for this study, when tracheal/bronchial cells are more likely to be exposed to flavoured-air under “real-world” usage scenarios. Changing anaesthesia best-practice in Australia limits our ability to access tracheal cells, while intrinsic similarities between the nasal and bronchial epithelium mean that our results are clinically valid (Kicic et al. 2020). Our balanced but modest sample (four female, four male donors) was underpowered for formal analysis of sex-specific effects. Although an exploratory analysis did not alter the primary findings, nominal sex-associated differences in apical protein release and TEER were observed; these warrant confirmation in adequately powered, sex-stratified studies of susceptibility to flavoured-air exposure. Additionally, our chemical analysis of flavoured-air cores was a preliminary, semi-quantitative analysis. No internal or external calibration standards were employed; therefore, absolute concentrations cannot be reported. Non-volatile and high-molecular-weight components of vapour that may not be well-resolved under the chromatographic conditions employed are not captured in this analysis. Thus the correlations between product chemistry and health outcomes should be interpreted cautiously given the constraints of six products, semi‑quantitative chemical data, compositional closure effects, and co‑variation among analytes within essential oil blends.

In conclusion, flavoured-air inhalers containing essential oils caused significant epithelial barrier dysfunction, inflammatory dysregulation, and structural damage in healthy human nasal airway epithelial cells after just one-hour of exposure. Our findings contradict manufacturer safety claims and raise concerns about the appropriateness of marketing these devices in the absence of adequate safety evaluation. More detailed toxicological assessment of these products is urgently needed before they can be recommended for inhalation use.

## Supplementary Information

Below is the link to the electronic supplementary material.

Supplementary Material 1

Supplementary Material 2

Supplementary Material 3

Supplementary Material 4

Supplementary Material 5

Supplementary Material 6

Supplementary Material 7

## Data Availability

Raw data can be made available upon reasonable request to corresponding author.

## Declarations

### Cell line declaration

Primary nasal airway epithelial cells were obtained via nasal brushing from participants in the CONQUER cohort study (RGS0000004379) under ethics approval RGS0000004379.
