# Supplementary material for: Toxic in vitro effects of flavoured-air inhalers containing essential oils in differentiated human nasal epithelial cells at air–liquid interface
Source: Arch Toxicol. 2026 Aug 12;100(12):5611–23. doi: 10.1007/s00204-026-04521-w (PMC13748146; doi:10.1007/s00204-026-04521-w)
Supplement: Supplementary file 1 [file 204_2026_4521_MOESM1_ESM.pdf]

Archives of Toxicology

Toxic *in vitro* effects of flavoured-air inhalers containing essential oils in differentiated human nasal epithelial cells at air-liquid interface.

AN Larcombe[1,2], EE Catchpole[2], P Hopper[3], S. Stanley[3], A Kicic[2,4,5], KR Landwehr[1,2] on behalf of CONQUER[5].

[1]*Occupation, Environment and Safety*, *School of Population Health, Curtin University*

[2]*Wal-yan Respiratory Research Centre, The Kids Research Institute Australia*

[3]*WA Organic & Isotope Geochemistry Centre, School of Earth and Planetary Sciences, Faculty of Science and Engineering, Curtin University, Bentley, Western Australia, Australia*

[4]*Department of Respiratory and Sleep Medicine, Perth Children's Hospital, Nedlands, Western Australia, Australia*

[5]*Centre for Cell Therapy and Regenerative Medicine, School of Medicine and Pharmacology, The University of Western Australia and Harry Perkins Institute of Medical Research, Perth, Western Australia, Australia*

Address for correspondence:
Alexander Larcombe,
Email: alexander.larcombe@curtin.edu.au

Supplementary Methods

Chemical Analyses of Flavoured-Air

Six essential oil-infused cores (one of each flavour) from commercially available flavoured-air devices were analysed alongside a methanol solvent blank. If necessary, cores were removed from their device housing. The fluid from the infused core material (medical-grade polyester fibre for Füm and Lio; absorbent wick material for Cigtrus) was recovered by placing the core in a glass vial and collecting the freely available fluid. Samples were prepared to 1 mg/mL in HPLC-grade methanol. A solvent blank consisting of neat methanol was prepared and analysed under identical conditions to allow identification and exclusion of solvent-related artefacts, column bleed, and system contaminants.

Analysis was performed on an Agilent 7890A gas chromatograph coupled to an Agilent 5975C Mass spectrometer, equipped with a HP-INNOWax Polyethylene Glycol column (30 m × 250 µm × 0.25 µm). A 1 µL sample was injected into a split/splitless inlet operating in split mode (split ratio 100:1, inlet temperature 270°C, He carrier gas flow 1.1 mL min^{-1}). The GC oven was initially held at 40°C for 2 minutes following injection, then heated to 260°C at 15°C min^{-1}, where it was held for 3.333 minutes. The mass spectrometer was operated in electron ionisation (EI) mode at 70 eV. Full-scan acquisition was employed over a range of 40–550 m/z.

Compound identification was performed by automated spectral matching against the NIST 2017 Mass Spectral Library (NIST17.L), and retention indices. Relative proportions of each identified compound were calculated as the ratio of individual peak area to the total integrated peak area for all identified compounds within each sample.

Tissue Culture

Primary airway epithelial cell cultures and air–liquid interface (ALI) differentiated models were prepared as previously reported (Martinovich et al. 2017). Cultures were maintained aseptically at 37°C in 5% CO^2/95% air. All cells were mycoplasma-negative. Cells were

expanded weekly in Corning T75 tissue culture flasks (CLS430720, Corning®, MERCK, NSW, AUS) and, in every case, used for differentiation before the third passage. For differentiation, cells were plated at 250,000 cells per membrane in PneumaCult ExPlus (Stemcell, Australia) onto Transwell inserts (Corning® Transwell, 12 mm, 0.4 μm pore polyester membrane, MERCK, NSW, AUS), allowed to reach confluence over ~3 days, then air-lifted and the basal medium replaced with PneumaCult ALI (Stemcell, Australia). Cultures were differentiated for at least 28 days prior to use in experiments. Transepithelial resistance (TEER) was measured weekly, with the final measurement taken immediately before exposures; cultures showing TEER values that deviated substantially from the subject mean were excluded. Media were refreshed before all exposures.

Exposure Protocol

Cell cultures were randomised in layout on the culture plate. The flavoured air was produced by passing medical air from a regulated source through the inhaler at a rate of 1 L/min such that the flavoured air exited the mouthpiece, through a short length of polyethylene tube and into the 1000 mL exposure chambers (Figure 1). Exposure chambers were placed into a TC-18UG-12/24 portable incubator (Waeco, Germany) maintained at 37°C for the 1-hour exposure period. Chambers were thoroughly cleaned and dried between exposures. After exposure, cell cultures were placed in an incubator set at 37°C in an atmosphere of 5% CO_2/95% air under aseptic conditions.

Lactate Dehydrogenase and Protein

After being left to rest for 24 hours, transepithelial electrical resistance was measured using an Epithelial Volt/Ohm (TEER) Meter (EVOM™ MANUAL with 24-well electrode, MERCK, Australia) to assess model integrity. Basal supernatant and apical wash (0.2 mL media wash of the apical surface) were collected. Toxicity was determined by measuring lactate dehydrogenase (LDH) release using the CytoTox 96 assay (Promega, Sydney, NSW, Australia,

#G1780) according to the supplied protocol and as previously described (Looi et al. 2016). Protein concentration was assessed using a Pierce™ BCA protein assay kit (Thermofisher Scientific, MA, USA) (Looi et al. 2016).

Mediators

Mediator levels were measured using commercially available kits (Bio-Rad 27plex human cytokine kit; Bio-Rad, CA, USA) and accompanying software (Bio-Plex Manager, v6.1.1, Bio-Rad, Tokyo, Japan). Data were processed using custom-written Python software which (i) removed individual data points that were >3 SD from the mean for that treatment, (ii) replaced any measurements below the limit of detection with a value of half the lowest standard for that analyte, and (iii) replaced any measurements above the limit of detection with the highest standard for that analyte.

Histology

Inserts were fixed in 10% formalin for 30 minutes, stored in 100% ethanol until all samples were collected, then embedded in paraffin. Sections 5 µm thick were cut and stained with Alcian Blue and counterstained with Neutral Red for visualisation of mucous-producing cells and other structures. Sections were scanned at 40× magnification using a SLIDEVIEW VS200 research slide scanner (Olympus) and measurements conducted with QuPath v0.6.0. Custom Groovy scripts were used to detect, count and measure the area of mucous-producing cells. Epithelial thickness was measured at three standardised intervals (¼, ½, and ¾) along the basement membrane, and the number of cell layers was manually counted at each point. Cilia density was scored on a 1–3 scale (1 = none, 2 = <50%, 3 = >50% coverage of luminal surface). The number of mucous-producing cells was normalised to the length of the basement membrane.

Statistics

LDH, BCA and TEER data were analysed using a linear mixed-effects model with treatment as a fixed effect and patient as a random effect. Pairwise comparisons between treatments were derived from the model parameter estimates. Group means and summary statistics were calculated using Python (pandas v2.3.3 and statsmodels v0.14.5) libraries.

Bioplex data were analysed using custom Python code (statsmodels v0.14.5, pandas v2.3.3, numpy v1.26.4). For each analyte, a linear mixed-effects model with treatment as a fixed effect and patient as a random intercept was fitted. Where the mixed-effects model did not converge, alternative model types were tested, including generalised linear models with Gaussian (identity and log link) or Gamma (log link) families, and ordinary least squares (OLS) regression. The best-fitting model was selected based on the highest coefficient of determination (R^2). For mixed-effects models, intraclass correlation coefficients (ICC) were calculated to quantify the proportion of variance attributable to between-patient variability. Pairwise comparisons between treatment groups were derived from the fitted models, with each treatment serving as the reference level in turn. Statistical significance was assessed using t-tests or z-tests as appropriate for the model type, with significance level set at $p < 0.05$.

Multivariate integration of chemical and biological data

The relative abundance (% TIC peak area) of each GC-MS analyte was compiled for the six products. Of 29 detected analytes, 12 present in two or more products were retained; analytes unique to a single product were excluded as they are collinear with product identity. The Air control was excluded as it contains no volatile constituents. Product-level means for 47 endpoints were used: TEER; LDH (apical and basal); BCA total protein (apical and basal); 16 apical and 20 basal Bioplex analytes above the limit of detection (consistent with the exclusion criteria applied in the primary Bioplex analyses; see main text); and six histological parameters (blue cells per basement membrane length, mean blue cell area, percentage mucous area,

epithelial thickness, number of cell layers, cilia score). Both matrices were centred and standardised. Because chemical variables (12) exceeded observations (6), PCA was applied to the chemical matrix; the first two PCs (87.2% of variance) served as constraining variables. PC1 (51.9%) separated sesquiterpene/cinnamaldehyde-rich products from menthol-dominated formulations; PC2 (35.3%) distinguished products higher in menthone, menthyl acetate, and pulegone. Significance was assessed by permutation test (9,999 permutations). Results are presented as a triplot of site scores (products), species scores (biological endpoints), and environmental correlations (chemical analytes). Pairwise Spearman r_s values were calculated between each chemical analyte and biological endpoint ($n = 6$ products; critical $|r_s| \approx 0.829$ for $p < 0.05$). No multiple comparisons correction was applied, consistent with the exploratory nature of the analysis. All analyses were performed using Python 3.12.3 and the following dependencies: NumPy 2.4.3; SciPy 1.17.1 (scipy.stats.spearmanr); scikit-learn 1.8.0 (PCA, StandardScaler); Matplotlib 3.10.8.

References

Looi K, Troy NM, Garratt LW, et al. (2016) Effect of human rhinovirus infection on airway epithelium tight junction protein disassembly and transepithelial permeability. Experimental Lung Research 42(7):380-395

Martinovich KM, Iosifidis T, Buckley AG, et al. (2017) Conditionally reprogrammed primary airway epithelial cells maintain morphology, lineage and disease specific functional characteristics. Scientific reports 7(1):17971
