# Supplementary material for: Toxic in vitro effects of flavoured-air inhalers containing essential oils in differentiated human nasal epithelial cells at air–liquid interface
Source: Arch Toxicol. 2026 Aug 12;100(12):5611–23. doi: 10.1007/s00204-026-04521-w (PMC13748146; doi:10.1007/s00204-026-04521-w)
Supplement: Supplementary file 2 [file 204_2026_4521_MOESM2_ESM.pdf]

Archives of Toxicology

Toxic *in vitro* effects of flavoured-air inhalers containing essential oils in differentiated human nasal epithelial cells at air-liquid interface.

AN Larcombe[1,2], EE Catchpole[2], P Hopper[3], S. Stanley[3], A Kicic[2,4,5], KR Landwehr[1,2] on behalf of CONQUER[5].

[1]*Occupation, Environment and Safety, School of Population Health, Curtin University*

[2]*Wal-yan Respiratory Research Centre, The Kids Research Institute Australia*

[3]*WA Organic & Isotope Geochemistry Centre, School of Earth and Planetary Sciences, Faculty of Science and Engineering, Curtin University, Bentley, Western Australia, Australia*

[4]*Department of Respiratory and Sleep Medicine, Perth Children's Hospital, Nedlands, Western Australia, Australia*

[5]*Centre for Cell Therapy and Regenerative Medicine, School of Medicine and Pharmacology, The University of Western Australia and Harry Perkins Institute of Medical Research, Perth, Western Australia, Australia*

Address for correspondence:

Alexander Larcombe,

Email: alexander.larcombe@curtin.edu.au

Supplementary Table 1 – Final number of cultures included per treatment and endpoint after exclusions. Values are the number of ALI inserts (two per donor per condition) contributing to each endpoint; “Donors” is the number of contributing donors. TEER was based on seven donors (one donor's measurements excluded as poor quality); histology was scored on one section per donor per treatment, with two donors excluded for poor-quality sections. *Mediator (Bio-Plex) inclusion was 14–16 per treatment and varied by analyte after the detection-limit and outlier criteria (Online Resources 6 and 7). FCM, Füm Crisp Mint; FMP, Füm Maple Pepper; LCS, Lio Cinnamon Spice; LMM, Lio Minty Magic; CGF, Cigtrus Grapefruit; CIP, Cigtrus Icy Peppermint.

Endpoint	Air	FCM	FMP	LCS	LMM	CGF	CIP	Donors
**Apical LDH**	16	16	15	16	16	16	16	8
**Basal LDH**	16	16	15	16	16	16	16	8
**Apical BCA protein**	16	16	16	16	16	16	16	8
**Basal BCA protein**	16	16	16	16	16	16	16	8
**TEER**	14	13	13	14	14	14	14	7
**Mediators (apical)***	–	–	–	–	–	–	–	8
**Mediators (basal)***	–	–	–	–	–	–	–	8
**Histology**	6	5	6	6	6	6	5	6
