# Supplementary material for: Toxic in vitro effects of flavoured-air inhalers containing essential oils in differentiated human nasal epithelial cells at air–liquid interface
Source: Arch Toxicol. 2026 Aug 12;100(12):5611–23. doi: 10.1007/s00204-026-04521-w (PMC13748146; doi:10.1007/s00204-026-04521-w)
Supplement: Supplementary file 3 [file 204_2026_4521_MOESM3_ESM.pdf]

Archives of Toxicology

Toxic *in vitro* effects of flavoured-air inhalers containing essential oils in differentiated human nasal epithelial cells at air-liquid interface.

AN Larcombe[1,2], EE Catchpole[2], P Hopper[3], S. Stanley[3], A Kicic[2,4,5], KR Landwehr[1,2] on behalf of CONQUER[5].

[1]*Occupation, Environment and Safety, School of Population Health, Curtin University*

[2]*Wal-yan Respiratory Research Centre, The Kids Research Institute Australia*

[3]*WA Organic & Isotope Geochemistry Centre, School of Earth and Planetary Sciences, Faculty of Science and Engineering, Curtin University, Bentley, Western Australia, Australia*

[4]*Department of Respiratory and Sleep Medicine, Perth Children's Hospital, Nedlands, Western Australia, Australia*

[5]*Centre for Cell Therapy and Regenerative Medicine, School of Medicine and Pharmacology, The University of Western Australia and Harry Perkins Institute of Medical Research, Perth, Western Australia, Australia*

Address for correspondence:

Alexander Larcombe,

Email: alexander.larcombe@curtin.edu.au

Supplementary Table 2 – Sensitivity analyses for the primary endpoints, using linear mixed-effects models with patient as a random intercept. (a) Nested model: the treatment factor reparameterised as flavour nested within brand. The within-brand flavour effect compares the full six-product model with a brand-only model; the brand main effect compares the brand-only model with an intercept-only model (likelihood-ratio tests). (b) Sex analysis: sex main effect (from a model including sex as a covariate) and sex x treatment interaction (likelihood-ratio test); n = 4 female / 4 male donors (TEER, 4 female / 3 male). All p-values are nominal and uncorrected for multiple testing.

(a) Nested model (flavour within brand)

**Endpoint**	**Within-brand flavour effect (LRT)**	**Brand main effect (LRT)**
**TEER**	$\chi^2 = 8.59$, df = 3, p = 0.035	$\chi^2 = 18.52$, df = 3, p < 0.001
**Apical BCA protein**	$\chi^2 = 8.85$, df = 3, p = 0.031	$\chi^2 = 13.70$, df = 3, p = 0.003
**Basal BCA protein**	$\chi^2 = 9.45$, df = 3, p = 0.024	$\chi^2 = 17.22$, df = 3, p < 0.001
**Apical LDH**	$\chi^2 = 4.22$, df = 3, p = 0.239	$\chi^2 = 2.89$, df = 3, p = 0.409
**Basal LDH**	$\chi^2 = 8.00$, df = 3, p = 0.046	$\chi^2 = 3.53$, df = 3, p = 0.317

(b) Sex covariate and sex × treatment interaction

**Endpoint**	**Sex main effect**	**Sex × treatment interaction (LRT)**
**TEER (4F/3M)**	p = 0.007	$\chi^2 = 11.10$, df = 6, p = 0.085
**Apical BCA protein**	p = 0.007	$\chi^2 = 25.46$, df = 6, p < 0.001
**Basal BCA protein**	p = 0.084	$\chi^2 = 9.06$, df = 6, p = 0.170
**Apical LDH**	p = 0.338	$\chi^2 = 5.18$, df = 6, p = 0.522
**Basal LDH**	p = 0.456	$\chi^2 = 2.07$, df = 6, p = 0.913
