# Supplementary material for: Toxic in vitro effects of flavoured-air inhalers containing essential oils in differentiated human nasal epithelial cells at air–liquid interface
Source: Arch Toxicol. 2026 Aug 12;100(12):5611–23. doi: 10.1007/s00204-026-04521-w (PMC13748146; doi:10.1007/s00204-026-04521-w)
Supplement: Supplementary file 4 [file 204_2026_4521_MOESM4_ESM.pdf]

Archives of Toxicology

Toxic *in vitro* effects of flavoured-air inhalers containing essential oils in differentiated human nasal epithelial cells at air-liquid interface.

AN Larcombe[1,2], EE Catchpole[2], P Hopper[3], S. Stanley[3], A Kicic[2,4,5], KR Landwehr[1,2] on behalf of CONQUER[5].

[1]*Occupation, Environment and Safety, School of Population Health, Curtin University*

[2]*Wal-yan Respiratory Research Centre, The Kids Research Institute Australia*

[3]*WA Organic & Isotope Geochemistry Centre, School of Earth and Planetary Sciences, Faculty of Science and Engineering, Curtin University, Bentley, Western Australia, Australia*

[4]*Department of Respiratory and Sleep Medicine, Perth Children's Hospital, Nedlands, Western Australia, Australia*

[5]*Centre for Cell Therapy and Regenerative Medicine, School of Medicine and Pharmacology, The University of Western Australia and Harry Perkins Institute of Medical Research, Perth, Western Australia, Australia*

Address for correspondence:

Alexander Larcombe,

Email: alexander.larcombe@curtin.edu.au

Supplementary Table 3 – Chemical overlap among the six flavoured-air products. Presence (✓) or absence (blank) of each GC-MS-detected compound in each product; shaded cells are coloured by brand (Füm, Lio, Cigtrus) and products are ordered by hierarchical clustering. Compounds (rows) are ordered by the number of products in which they were detected (n, right-hand column), from most to least shared. Only menthol and caryophyllene were detected in all six products. Compounds marked with an asterisk were provisional identifications (weak spectral match). FCM, Füm Crisp Mint; FMP, Füm Maple Pepper; LCS, Lio Cinnamon Spice; LMM, Lio Minty Magic; CGF, Cigtrus Grapefruit; CIP, Cigtrus Icy Peppermint.

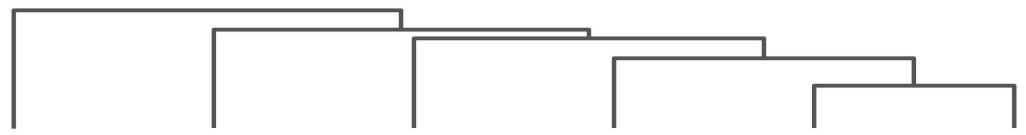

**Compound**	**CGF**	**CIP**	**FMP**	**LCS**	**FCM**	**LMM**	**n**
Caryophyllene	✓	✓	✓	✓	✓	✓	6
Menthol	✓	✓	✓	✓	✓	✓	6
Isomenthone		✓	✓	✓	✓	✓	5
Menthone		✓	✓	✓	✓	✓	5
Eucalyptol			✓	✓	✓	✓	4
Menthyl acetate			✓	✓	✓	✓	4
Pulegone			✓	✓	✓	✓	4
Carvone				✓		✓	2
Caryophyllene oxide			✓		✓		2
Cinnamaldehyde			✓	✓			2
Linalool	✓		✓				2
α-Terpineol	✓		✓				2
Unknown (unidentified)			✓		✓		2
Acetic acid, cinnamyl ester				✓			1
Anethole				✓			1
Benzyl benzoate				✓			1

Cadinene isomer*			√		1
Cinnamaldehyde, o-methoxy-			√		1
Cinnamyl alcohol				√	1
Citral			√		1
Copaene			√		1
D-Limonene	√				1
Eugenol			√		1
Glycerol tricaprylate		√			1
Humulene			√		1
Octanoic acid, methyl ester	√				1
Triethylene glycol monobutyl ether				√	1
Vanillin		√			1
β-Bisabolene			√		1
γ-Muurolene*			√		1
