# Supplementary material for: Toxic in vitro effects of flavoured-air inhalers containing essential oils in differentiated human nasal epithelial cells at air–liquid interface
Source: Arch Toxicol. 2026 Aug 12;100(12):5611–23. doi: 10.1007/s00204-026-04521-w (PMC13748146; doi:10.1007/s00204-026-04521-w)
Supplement: Supplementary file 5 [file 204_2026_4521_MOESM5_ESM.pdf]

Archives of Toxicology

Toxic *in vitro* effects of flavoured-air inhalers containing essential oils in differentiated human nasal epithelial cells at air-liquid interface.

AN Larcombe[1,2], EE Catchpole[2], P Hopper[3], S. Stanley[3], A Kicic[2,4,5], KR Landwehr[1,2] on behalf of CONQUER[5].

[1] *Occupation, Environment and Safety, School of Population Health, Curtin University*

[2] *Wal-yan Respiratory Research Centre, The Kids Research Institute Australia*

[3] *WA Organic & Isotope Geochemistry Centre, School of Earth and Planetary Sciences, Faculty of Science and Engineering, Curtin University, Bentley, Western Australia, Australia*

[4] *Department of Respiratory and Sleep Medicine, Perth Children's Hospital, Nedlands, Western Australia, Australia*

[5] *Centre for Cell Therapy and Regenerative Medicine, School of Medicine and Pharmacology, The University of Western Australia and Harry Perkins Institute of Medical Research, Perth, Western Australia, Australia*

Address for correspondence:

Alexander Larcombe,

Email: alexander.larcombe@curtin.edu.au

Supplementary Table 4 – GCMS analysis of volatile and semi-volatile organic compounds in six flavoured-air products. Values are percent relative abundance.

**Compound**	**Füm Crisp Mint (FCM)**	**Füm Maple Pepper (FMP)**	**Lio Cinnamon Spice (LCS)**	**Lio Minty Magic (LMM)**	**Cigtrus Grapefruit (CGF)**	**Cigtrus Icy Peppermint (CIP)**	**GHS Hazard Statements from PubChem**
Acetic acid, cinnamyl ester			1.99%				*Not classified*
Anethole			0.58%				H317
Benzyl Benzoate			16.77%				H302, H411
Cadinene isomer (?)		1.89%					*Not classified*
Carvone			0.60%	0.84%			H317
Caryophyllene	3.39%	13.32%	2.52%	4.17%	0.66%	0.91%	H304, H317
Caryophyllene oxide	1.69%	2.64%					H315, H319, H411
Cinnamaldehyde		20.02%	6.93%				H317
Cinnamaldehyde, o-methoxy-		1.90%					*Not classified*
Cinnamyl alcohol			0.97%				H302, H317
Citral		0.83%					H315, H317
Copaene		1.28%					H226, H304, H315
D-Limonene					2.26%		H226, H304, H315, H317, H400, H412
Eucalyptol	0.53%	1.40%	0.41%	0.34%			H226, H317
Eugenol		14.62%					H302, H315, H317, H319
Glycerol tricaprylate						18.52%	*Not classified*
Humulene		1.15%					H315, H319, **H335**
Isomenthone	3.04%	0.75%	3.91%	5.26%		1.21%	H315, H317
Linalool		1.22%			0.46%		H317
Menthol	66.67%	15.63%	40.98%	71.77%	94.98%	75.09%	H315, H319
Menthone	14.67%	3.66%	7.99%	10.60%		3.47%	H226, H315, H319, H412
Menthyl acetate	8.06%	2.29%	3.89%	6.23%			H411
Octanoic acid, methyl ester					1.04%		H317
Pulegone	0.98%	0.61%	0.48%	0.78%			H302

Triethylene glycol monobutyl ether			13.99%				H318
Vanillin						0.81%	H317, H319
α-Terpineol		2.45%			0.59%		H315, H319
β-Bisabolene		1.97%					H304, H315, H317, **H332**, H400, H410, H411
γ-Muurolene (?)		0.82%					*Not classified*
Unknown	0.98%	9.57%					*N/A*

* GHS Hazard Statements were obtained from PubChem. Codes in **bold** refer to harmful effects on the respiratory system. H226 – Flammable liquid and vapour, H302 – Harmful if swallowed, H304 – May be fatal if swallowed and enters airways, H315 – Causes skin irritation, H317 – May cause an allergic skin reaction, H318 – Causes serious eye damage, H319 – Causes serious eye irritation, **H332 – Harmful if inhaled**, **H335 – May cause respiratory irritation**, H400 – Very toxic to aquatic life, H410 – Very toxic to aquatic life with long lasting effects, H411 – Toxic to aquatic life with long lasting effects, H412 – Harmful to aquatic life with long lasting effects. (?) refers to a weak spectral match; identification is provisional.
