# Supplementary material for: Toxic in vitro effects of flavoured-air inhalers containing essential oils in differentiated human nasal epithelial cells at air–liquid interface
Source: Arch Toxicol. 2026 Aug 12;100(12):5611–23. doi: 10.1007/s00204-026-04521-w (PMC13748146; doi:10.1007/s00204-026-04521-w)
Supplement: Supplementary file 6 [file 204_2026_4521_MOESM6_ESM.pdf]

Archives of Toxicology

Toxic *in vitro* effects of flavoured-air inhalers containing essential oils in differentiated human nasal epithelial cells at air-liquid interface.

AN Larcombe[1,2], EE Catchpole[2], P Hopper[3], S. Stanley[3], A Kicic[2,4,5], KR Landwehr[1,2] on behalf of CONQUER[5].

[1]*Occupation, Environment and Safety, School of Population Health, Curtin University*

[2]*Wal-yan Respiratory Research Centre, The Kids Research Institute Australia*

[3]*WA Organic & Isotope Geochemistry Centre, School of Earth and Planetary Sciences, Faculty of Science and Engineering, Curtin University, Bentley, Western Australia, Australia*

[4]*Department of Respiratory and Sleep Medicine, Perth Children's Hospital, Nedlands, Western Australia, Australia*

[5]*Centre for Cell Therapy and Regenerative Medicine, School of Medicine and Pharmacology, The University of Western Australia and Harry Perkins Institute of Medical Research, Perth, Western Australia, Australia*

Address for correspondence:

Alexander Larcombe,

Email: alexander.larcombe@curtin.edu.au

Supplementary Table 5 – Mean mediator levels in the apical compartment. Data are mean±SD. a = different to air, b = different to FCM, c = different to FMP, d = different to LCS, e = different to LMM, f = different to CGF, g = different to CIP. N = 14-16.

Mediator	Air	FCM	FMP	LCS	LMM	CGF	CIP
IL-1ra	180.9±109.4	344.8±598.8[g]	324.4±324.3	163.4±84.9	225.8±171.4	165.4±99.3	145.7±94.6[b]
IL-5	30.8±11.3[c,f,g]	28.3±13.7[f]	20.6±11.2[a,d,e]	31.5±12.6[c,f,g]	35.1±20.8[c,f,g]	14.1±12.8[a,b,d,e]	21.3±10.3[a,d,e]
IL-6	35.1±29.9	22.5±24.9[f]	20.8±19.1[f]	32.6±28.4	29.1±36.4[f]	47.2±48.9[b,c,e,g]	27.8±26.4[f]
IL-7	155.1±90[d,f]	130.3±73.9[c,d,f]	179.9±93.2[b,g]	203.7±111.5[a,b,e,g]	153.0±101.9[d,f]	202.5±91.7[a,b,e,g]	118.9±73.9[c,d,f]
IL-8	5296.2±3715.2[e]	6143.3±3595.5[e]	5410.7±3341.8[e]	5256.9±3138.1[e]	3268.3±1609.3[a,b,c,d]	4984.5±2901.1	5159.9±3707.3
IL-9	55.7±17.2	50.9±17.2[d]	51.6±12.4	58.4±13.5[b,g]	56.1±18.5	51.9±19.0	51.1±18.0[d]
Eotaxin	0.69±0.41[c,g]	0.47±0.40[e,g]	0.35±0.21[a,e,f]	0.58±0.36[e,g]	0.84±0.68[b,c,d,g]	0.61±0.49[c,g]	0.22±0.16[a,b,d,e,f]
G-CSF	40.4±39.2	27.6±27.2	26.8±18.6	44.8±41.9	52.8±103.9	49.1±69.7	34.5±36.4
GM-CSF	4.65±4.42	4.40±4.61	2.03±2.21[d,e,f]	6.09±5.92[c]	5.80±6.13[c]	6.40±6.20[c]	4.59±5.18
IFNγ	17.7±10.5	23.7±35.1	25.4±19.7	17.6±10.3	21.4±12.5	20.1±13.6	15.8±9.9
IP-10	1462.3±966.4[d,f]	1041.5±529.7[d,f,g]	1499.7±627.4[d,f]	2224.4±1108.3[a,b,c,e]	1360.4±571.6[d,f]	2083.1±1222.0[a,b,c,e]	1697.2±965.2[b]
MCP-1	49.9±47.9[f]	48.9±65.6[f]	63.5±79.6	52.2±46.6	50.4±52.9[f]	81.2±94.1[a,b,e,g]	53.8±55.0[f]
MIP-1α	0.19±0.13[b,c]	0.09±0.07[a,d,e,f]	0.07±0.05[a,d,e,f,g]	0.17±0.12[b,c]	0.18±0.05[b,c]	0.19±0.09[b,c]	0.15±0.12[c]
MIP-1β	16.9±5.4	16.9±5.9	16.9±3.9	18.9±4.7	16.9±6.3	18.4±6.7	16.8±6.3
RANTES	22.3±13.5[e]	21.7±16.0[e]	19.9±11.4[e]	24.8±15.5	29.7±24.9[a,b,c,f]	19.8±18.2[e]	21.1±19.8
TNF-α	80.3±39.5[e]	68.2±45.3[e,f]	47.4±34.1[d,e,f]	83.5±50.1[c,e]	116.5±101.3[a,b,c,d,g]	101.1±70.9[b,c]	77.2±65.4[e]
