# Supplementary material for: Toxic in vitro effects of flavoured-air inhalers containing essential oils in differentiated human nasal epithelial cells at air–liquid interface
Source: Arch Toxicol. 2026 Aug 12;100(12):5611–23. doi: 10.1007/s00204-026-04521-w (PMC13748146; doi:10.1007/s00204-026-04521-w)
Supplement: Supplementary file 7 [file 204_2026_4521_MOESM7_ESM.pdf]

Archives of Toxicology

Toxic *in vitro* effects of flavoured-air inhalers containing essential oils in differentiated human nasal epithelial cells at air-liquid interface.

AN Larcombe[1,2], EE Catchpole[2], P Hopper[3], S. Stanley[3], A Kicic[2,4,5], KR Landwehr[1,2] on behalf of CONQUER[5].

[1]*Occupation, Environment and Safety, School of Population Health, Curtin University*

[2]*Wal-yan Respiratory Research Centre, The Kids Research Institute Australia*

[3]*WA Organic & Isotope Geochemistry Centre, School of Earth and Planetary Sciences, Faculty of Science and Engineering, Curtin University, Bentley, Western Australia, Australia*

[4]*Department of Respiratory and Sleep Medicine, Perth Children's Hospital, Nedlands, Western Australia, Australia*

[5]*Centre for Cell Therapy and Regenerative Medicine, School of Medicine and Pharmacology, The University of Western Australia and Harry Perkins Institute of Medical Research, Perth, Western Australia, Australia*

Address for correspondence:

Alexander Larcombe,

Email: alexander.larcombe@curtin.edu.au

Supplementary Table 6 – Mean mediator levels in the basal compartment. Data are mean±SD. a = different to air, b = different to FCM, c = different to FMP, d = different to LCS, e = different to LMM, f = different to CGF, g = different to CIP. N = 14-16.

Mediator	Air a	FCM b	FMP c	LCS d	LMM e	CGF f	CIP g
IL-1ra	122.7±69.1	159.3±235.5^{f,g}	127.0±128.3	96.8±58.9	93.7±49.2	88.1±76.4^{b}	85.1±48.9^{b}
IL-4	1.68±0.80^{c,d,f,g}	1.59±0.44^{c,d,g}	1.11±0.64^{a,b,e}	1.13±0.48^{a,b,e}	1.65±0.62^{c,d,g}	1.24±0.54^{a}	0.86±0.76^{a,b,e}
IL-5	40.6±23.0^{c,d,f,g}	33.1±8.6^{f,g}	28.5±9.8^{a,f}	30.1±9.1^{a,f}	33.9±8.3^{f,g}	13.0±9.1^{a,b,c,d,e,g}	24.6±11.7^{a,b,e,f}
IL-6	13.5±19.8	12.7±23.2	10.4±16.4	12.4±21.5	6.3±8.9	12.5±19.4	5.7±8.1
IL-8	2815.5±3037.5	3725.9±2592.4^{e,f}	2911.8±2365.8	3207.3±3351.8	2318.8±1367.2^{b}	2241.3±1543.5^{b}	2559.3±2757.3
IL-9	42.1±15.8^{f,g}	40.0±13.8^{g}	36.1±11.1	40.6±14.1^{g}	35.1±10.3	34.2±19.6^{a}	32.4±17.0^{a,b,d}
IL-17	6.70±6.63^{f,g}	5.79±3.02^{f}	4.83±2.96	4.45±2.20	5.64±2.96^{f}	3.27±1.31^{a,b,e}	4.01±2.74^{a}
Eotaxin	0.92±0.60^{b,c,d,g}	0.65±0.35^{a}	0.54±0.40^{a}	0.62±0.26^{a}	0.74±0.33^{g}	0.68±0.28^{g}	0.42±0.38^{a,e,f}
FGF basic	670.9±160.1^{f,g}	673.9±226.8^{f,g}	572.6±146.6^{d,e}	682.5±127.9^{c,f,g}	759.4±160.6^{c,f,g}	528.2±177.6^{a,b,d,e}	482.7±105.0^{a,b,d,e}
G-CSF	28.8±26.3^{g}	36.9±40.2^{c,e,f,g}	20.2±19.6^{b}	23.9±22.2	18.1±6.6^{b}	21.0±17.6^{b}	17.8±17.3^{a,b}
GM-CSF	2.86±2.83^{c}	1.84±1.69	1.30±1.16^{a,f,g}	2.30±2.55	2.08±1.95	2.79±2.22^{c}	2.70±4.20^{c}
IFNγ	13.3±10.7^{d,g}	12.5±14.1	9.8±8.7	8.2±6.1^{a}	8.1±6.1	10.3±12.1	8.1±7.8^{a}
IP-10	474.5±531.4	678.7±884.3^{c,f}	329.9±229.1^{b,d}	763.7±893.6^{c,f}	623.4±640.2	371.5±341.1^{b,d}	582.3±732.5
MCP-1	42.7±63.6	41.6±62.9	44.5±76.0	43.5±68.4	47.5±95.4	65.4±102.4	48.2±73.2
MIP-1α	0.27±0.15^{c,d,e,f,g}	0.22±0.06	0.18±0.08^{a}	0.19±0.05^{a}	0.19±0.06^{a}	0.20±0.11^{a}	0.16±0.08^{a}
PDGF-bb	63.5±23.6^{c,g}	66.4±20.2^{c,g}	38.0±19.8^{a,b,d,e,f}	60.5±20.0^{c}	71.2±23.5^{c,g}	65.2±14.8^{c,g}	46.7±18.8^{a,b,e,f}
MIP-1β	11.2±4.5	11.2±4.5	10.3±3.5	11.6±4.8	10.3±3.4	11.4±7.1	9.6±5.5
RANTES	14.8±12.0	15.0±11.4	12.4±7.8	14.7±13.2	10.1±6.3	15.2±11.1	14.7±8.1
TNF-α	81.8±55.8^{c,d,g}	67.0±42.6	49.9±32.0^{a,e,f}	55.9±48.1^{a,f}	68.8±31.8^{c}	78.7±49.6^{c,d,g}	55.2±54.2^{a,f}
VEGF	441.1±292.5^{b,c,e,f,g}	374.7±268.5^{a,g}	320.4±233.5^{a,g}	370.5±267.0^{g}	330.6±239.8^{a,g}	319.5±186.0^{a,g}	226.9±184.2^{a,b,c,d,e,f}
